# Poster Session II - A286 REAL-WORLD OUTCOMES OF MIRIKIZUMAB THERAPY FOR ULCERATIVE COLITIS

**DOI:** 10.1093/jcag/gwaf042.286

**Published:** 2026-02-13

**Authors:** J S Moore, C McShane, R Milgrom, J Stempak, P Olivera, G Nguyen, A Weizman, K Croitoru, Z Gallinger, V W Huang, L Targownik, H Steinhart, S Lee, M Silverberg

**Affiliations:** Mount Sinai Hospital, Toronto, ON, Canada; Mount Sinai Hospital, Toronto, ON, Canada; Mount Sinai Hospital, Toronto, ON, Canada; Mount Sinai Hospital, Toronto, ON, Canada; Unity Health Toronto, Toronto, ON, Canada; Mount Sinai Hospital, Toronto, ON, Canada; Mount Sinai Hospital, Toronto, ON, Canada; Mount Sinai Hospital, Toronto, ON, Canada; Mount Sinai Hospital, Toronto, ON, Canada; Mount Sinai Hospital, Toronto, ON, Canada; Mount Sinai Hospital, Toronto, ON, Canada; Mount Sinai Hospital, Toronto, ON, Canada; Mount Sinai Hospital, Toronto, ON, Canada; Mount Sinai Hospital, Toronto, ON, Canada

## Abstract

**Background:**

Mirikizumab, an IL-23p19 inhibitor demonstrated efficacy in inducing and maintaining remission in ulcerative colitis (UC) within clinical trials. Real-world evidence remains limited.

**Aims:**

To evaluate the real-world effectiveness, durability, and safety of mirikizumab in UC patients.

**Methods:**

A retrospective cohort study was conducted at a tertiary referral center. Adult UC patients initiating mirikizumab with ≥12 weeks follow-up (January 2023-August 2025). Primary outcome was corticosteroid-free (CSF) clinical remission (partial Mayo ≤2, no subscore >1) at weeks 12, 28, and 52. Secondary outcomes included CSF clinical response (≥2-point and ≥30% reduction in partial Mayo), mucosal healing (endoscopic Mayo ≤1), and adverse events.

**Results:**

40 patients were included: 55% female, median age was 34 years (IQR 21-51), and baseline Mayo 6.85 (SD 2.36). Nearly all (39/40) had prior advanced therapy exposure. CSF clinical remission was achieved in 23% at week 12, 30% at week 28, and 35% at week 52. CSF clinical response was 35%, 48%, and 39% respectively. Allowing for steroids at 12 weeks, clinical remission rate was seen in 25% and response in 52% of patients. No significant changes in CRP or fecal calprotectin were observed. Mucosal healing rates were 27% (3/11) at 6 months and 25% (1/4) at 12 months. Extended induction was given to 47.5% (19/40), who had lower but non-significant odds of CSF remission at 6 and 12 months. Among 32.5% (13/40) with prior ustekinumab exposure, no differences in remission rates were observed versus those without prior exposure. No serious adverse events occurred.

**Conclusions:**

This real-world study of a highly treatment-experienced cohort shows CSF clinical remission rates at 12 weeks similar to phase III trials but lower rates at 52 weeks. Mirikizumab demonstrated acceptable safety. These findings support the effectiveness of mirikizumab for UC although further research is needed to identify optimal candidates.

A286 Table 1: Baseline Characteristics

**Funding Agencies:**

NoneNone

**Table:** 

Overall (n = 40^1^)	
Sex (male)	18 (45)
Age at initiation, years	34 [21 - 51]
UC Extent	
Proctitis	1 (2.5%)
Left-sided	8 (20%)
Pancolitis	31 (78%)
Previous UC-related hospitalization in the past year	17 (43%)
Number of previous advanced therapy classes	2.00 [1.00 - 3.00]
Systemic steroid at initiation	17 (43%)
Overall Mayo at baseline	6.85 (2.36)
Partial Mayo at baseline	4.38 (2.07)
Endoscopic Mayo at baseline	3.00 [2.00 - 3.00]
CRP at baseline (mg/L)	3 [1 - 8]
Fecal calprotectin at baseline (μg/g)	803 [560 - 1,786]
Albumin at baseline (g/L)	43.0 [42.0 - 45.0]

1 n (%); Median [IQR]; Mean (SD)

